# Development of the Competency Evaluation Scale for Clinical Nutritionists in China: A Delphi Study

**DOI:** 10.3390/nu16162593

**Published:** 2024-08-06

**Authors:** Ailin Zhou, Zhourong Li, Tiankun Wang, Rongxin Wu, Shuang Yang, Zumin Shi, Huan Zeng, Yong Zhao

**Affiliations:** 1 School of Public Health, Chongqing Medical University, Chongqing 400016, China; 2022120872@stu.cqmu.edu.cn (A.Z.); 2021120781@stu.cqmu.edu.cn (Z.L.); 2022120844@stu.cqmu.edu.cn (R.W.); 2022120852@stu.cqmu.edu.cn (S.Y.); huanzeng@cqmu.edu.cn (H.Z.); 2 Research Center for Medicine and Social Development, Chongqing Medical University, Chongqing 400016, China; 3 Research Center for Public Health Security, Chongqing Medical University, Chongqing 400016, China; 4 Nutrition Innovation Platform-Sichuan and Chongqing, Chongqing 400016, China; 5 Department of Clinical Nutrition, West China Second Hospital, Sichuan University, Chengdu 610041, China; wtk@scu.edu.cn; 6 Key Laboratory of Birth Defects and Related Diseases of Women and Children, Ministry of Education, Chengdu 610041, China; 7 Human Nutrition Department, College of Health Sciences, QU Health, Qatar University, Doha P.O. Box 2713, Qatar; zumin@qu.edu.qa; 8 Chongqing Key Laboratory of Child Nutrition and Health, Children’s Hospital of Chongqing Medical University, Chongqing 400014, China

**Keywords:** clinical nutritionists, competency, scale, Delphi, analytic hierarchy process

## Abstract

**Background:** Clinical nutritionists are responsible for nutritional therapy in clinical practice, which significantly enhances patients’ nutritional status. This study aims to develop and validate a competency evaluation scale to effectively assess the abilities of clinical nutritionists. **Methods:** The competency evaluation scale for clinical nutritionists was developed based on the iceberg model, utilizing literature review, semi-structured interviews, and the Delphi method. The weights of each indicator were calculated using the Analytic Hierarchy Process (AHP), and the validity and reliability of the scale were confirmed through questionnaire surveys. **Results:** The competency evaluation scale of clinical nutritionists comprised five primary indicators, twelve secondary indicators, and sixty-six tertiary indicators. The primary indicators, including professional theoretical knowledge, professional practical skills, humanistic practice ability, interpersonal communication ability, and professional development capability, have respective weights of 0.2168, 0.2120, 0.2042, 0.2022, and 0.1649. The Cronbach’s α coefficients of the five dimensions of the scale were 0.970, 0.978, 0.969, 0.962, and 0.947, respectively. The results of the Exploratory Factor Analysis showed that the prerequisites for factor analysis were satisfied. Additionally, Bartlett’s test of sphericity yielded a significance level of *p* < 0.001, confirming the scale’s reliability and validity. **Conclusions:** The competency evaluation scale for clinical nutritionists developed in this study is of high scientific reliability and validity, which provides assessment criteria for the training and assessment of clinical nutritionists.

## 1. Introduction

Nutrition is vital for human life and health, particularly for patients, as maintaining good nutritional status is crucial for disease recovery [1]. Numerous studies [2,3,4,5,6] have demonstrated that patients admitted to hospitals with nutritional risks or symptoms of malnutrition often experience poor clinical outcomes, such as disease onset, mortality, complications, prolonged hospital stays, readmission, and a significant decline in quality of life. Malnourished patients may experience a direct impact on their immune system [7] and treatment response, leading to an increased risk of infections, organ failure, and other serious complications [8]. Therefore, rational and standardized nutritional therapy can effectively reduce the risk of infection, improve clinical outcomes, and enhance the efficacy of medical treatment for patients [9,10]. In recent years, clinical nutrition has evolved as a technical tool to support clinical therapy, aiming to meet the unique nutritional needs of patients. The Guidelines for the Construction and Management of Clinical Nutrition Departments [11], released by the National Health Commission, state that clinical nutrition departments in hospitals across China have increasingly developed a variety of services, including nutritional screening and assessment, diagnosis, and treatment, as well as outreach and education. Therefore, clinical nutritionists [12], who are at the core of nutrition support therapy, play a crucial role in the overall recovery process by making professional decisions.

With the development of modern medicine, doctors or technicians who work in clinical environments related to nutrition and diet are called clinical nutritionists [13]. In the United States, individuals practicing as dietitians must pass a registration examination to obtain legal authorization for professional practice [14]. In contrast, China’s registered dietitian system began later than in the U.S. (1969) [15], and it was not until 2016 that the Chinese Nutrition Society formally established the Registered Dietitian Working Committee [16]. Furthermore, due to historical, policy, and other factors, China still faces numerous challenges in training clinical nutritionists [13]. The development of the clinical nutrition workforce remains a limiting factor for clinical nutrition departments, mainly due to lower educational achievements and professional titles among the professional and technical team members. Concurrently, there is a notable shortage of professionals with essential theoretical knowledge and practical skills in nutrition [17,18].

The competency refers to the ability to distinguish personal characteristics such as the performance level of the excellent and the average in a specific job and organizational environment, which can be used to measure the professionalism and work ability of clinical nutritionists [19]. Recently, foreign scholars have developed a competency evaluation tool for clinical nutritionists based on the classic iceberg model and onion model [20]. They utilized expert assessment, the Delphi method, and coordinated development among associations, among other methods. The Commission on Dietetic Registration [15] in the US, the Dietitians of Canada [21], the New Zealand Dietetic Association [22], and the European Federation of the Associations of Dietitians [23] have developed relevant competency evaluation tools. In addition, the Chinese Medical Doctor Association organized the first national examination for the Clinical Nutritionist Training and Assessment Program in 2015 [24]. While this has become the primary tool for evaluating the competency of clinical nutritionists in China, the examination content may not comprehensively address all competencies required for clinical nutritionists. Hence, China requires a more comprehensive evaluation tool for assessing the competency of clinical nutritionists. Currently, there has been thorough research on the competency evaluation index system for practicing physicians and nurses [25,26,27,28]. Nevertheless, a specialized competency assessment tool tailored for clinical nutritionists has not yet been developed. Therefore, the purpose of this study is to pioneer the construction of a competency evaluation scale for clinical nutritionists that is suitable for Chinese national conditions and status, aiming to effectively meet the urgent needs for the development and cultivation of clinical nutritional talents in the current period.

## 2. Methods

### 2.1. Literature Review and Semi-Structured Interviews

#### 2.1.1. Literature Review

This study systematically used the databases of the Web of Science, PubMed, ScienceDirect, Scopus, Google Scholar, China National Knowledge Infrastructure, and Wanfang, and mainly searched the Chinese and English literature on clinical nutritionists’ competency with the keywords of “clinical nutritionist”, “competency”, and “iceberg model”. According to the relevant textbooks on clinical nutrition and the Guidelines for the Construction and Management of Clinical Nutrition Departments in China [11], we developed the preliminary framework for evaluating the competency of clinical nutritionists.

McClelland first proposed the iceberg model of competency [19], which has since become widely adopted in competency studies of physicians and nurses [29,30,31]. The model divides competencies into those visible at the surface, such as knowledge and skills, and those latent below the surface, such as traits, social roles, and motivation. In the process of scale development, the iceberg model considers not only the penetrance characteristics of competencies but also delves into their intrinsic characteristics. Based on the penetrance characteristics that demonstrate the professional knowledge and skills required by clinical nutritionists, the model also explores their underlying personal values and other qualities. These factors significantly impact their work effectiveness and predict future work performance [32]. Therefore, using the iceberg model as the theoretical basis for scale development will allow for the consideration of the complexity and diversity of competency components, enhancing the accuracy and comprehensiveness of competency assessment.

#### 2.1.2. Semi-Structured Interviews

Semi-structured interviews were conducted with ten experts in the clinical nutrition field and experienced professionals to refine the competency evaluation scale for clinical nutritionists. The interview process was audio-recorded throughout, and then the recordings were converted into text for organization and analysis. The main interview questions included: (1) Please discuss your perspectives on Nutrition and Food Hygiene. (2) What are the daily tasks performed in the hospital’s clinical nutrition department? Describe the main steps of each task and the collaboration mechanism with other clinical departments. (3) What abilities and qualities do you believe students of nutrition and food hygiene should possess? (4) What specific competencies or qualities do you consider essential for clinical nutritionists working in hospitals? Finally, the competency evaluation scale of clinical nutritionists was developed, which included 5 primary indicators, 13 secondary indicators, and 48 tertiary indicators.

### 2.2. Delphi Expert Consultation

#### 2.2.1. Expert Panel

Using convenience sampling and a search for authors of relevant literature, 24 experts were chosen to fulfill the sample requirements for the Delphi method [33]. Inclusion criteria for the experts comprised the following: (1) possessing a bachelor’s degree or higher, (2) having a minimum of 5 years’ experience in clinical nutrition or nutrition management, (3) being presently employed in the field, and (4) providing informed consent and voluntarily participating in the study.

#### 2.2.2. Data Collection

The Delphi expert consultation process in this study comprised two rounds of email-based questionnaires administered in April and May 2022 [34]. Each consultation round spanned one week. The expert consultation includes four parts: (1) the purpose and significance of this research; (2) the basic information questionnaire for experts; (3) the expert evaluation form; and (4) a questionnaire on the level of authority of the experts. On the expert evaluation form, experts score the attribution and importance of each evaluation indicator. These are scored using binary classification, with “1” representing “yes” and “2” representing “no”. The degree of importance was rated on a 5-point Likert scale, ranging from “1” (unimportant) to “5” (very important). After the two rounds of expert consultation forms were recovered, the data were compiled and analyzed to determine the final competency evaluation indicators.

#### 2.2.3. Data Analysis

SPSS 26.0 and STATA 17.0 were used to analyze the data. The reliability of the experts was assessed in terms of the degree of activeness of experts, authority, and coordination. The response rate of expert consultation questionnaires was >70%, with experts considered to be highly active [35]. The composite reliability (Cr) of an expert is determined by the familiarity coefficient (Cs) and the judgement coefficient (Ca), Cr = (Cs + Ca)/2, and Cr ≥ 0.7 is considered to be more credible [27]. The degree of expert coordination is the Kendall’s coefficient, denoted by W, which ranges from 0 to 1, with a greater W indicating a higher degree of coordination [35].

Indicators meeting the following criteria will be retained: indicator selection rate >80%, a mean value of significance (Mean) > 4.0, standard deviation (SD) < 1.0, and a coefficient of variation (CV) < 0.2 [36]. Additionally, indicators will be added or modified based on suggestions from experts in response to open-ended questions and discussions within the subject group.

The Analytic Hierarchy Process (AHP) was employed in this study to calculate indicator weights and establish a hierarchical structural model. Satty’s 1–9 level scaling method was utilized to assign weights to each indicator. The AHP software yaahp 12.8 was used to calculate the weights, establish a hierarchical structure model, and carry out the consistency test. When the Consistency Ratio (CR) is ≤0.1, the hierarchical structure model is reasonable [37].

### 2.3. Validation of Scale Reliability and Validity

This study employed a cross-sectional research design and utilized convenience sampling to recruit clinical nutritionists from Sichuan and Chongqing as participants. A self-designed questionnaire based on the clinical nutritionists competency scale was distributed in October 2022 through the online platform of “Questionnaire Star”. The questionnaire comprised two sections: respondents’ basic information and the competency assessment. It took approximately ten minutes to complete. The purpose of this survey was stated at the beginning of the questionnaire, which was completed anonymously after they read the informed consent. Criteria for inclusion in the survey were the following: (1) clinical nutritionists in secondary or higher level hospitals with clinical nutrition departments in Sichuan and Chongqing and (2) those who gave informed consent. The exclusion criterion was (1) clinical nutritionists in training and internship.

Internal consistency reliability was evaluated using the Cronbach’s α coefficient in this study. Scale validity was assessed by testing survey reliability using AMOS 24.0 software. When the Cronbach’s α coefficient of the total scale is greater than 0.6, the questionnaire is considered to have good internal consistency [38]. Content validity was deemed satisfactory if an indicator was endorsed by over 80% of experts. Kaiser–Meyer–Olkin (KMO) and Bartlett’s test of sphericity were used for Exploratory Factor Analysis (EFA) to test the structural validity of the scale. The KMO value greater than 0.6 and the Bartlett’s test of sphericity result of *p* < 0.05 indicate that it is suitable for factor analysis [39].

## 3. Results

### 3.1. Basic Information on Experts

A total of 23 experts were selected for this study. The average age of the experts was 41.72 ± 5.71 years. Among them, twelve (52.2%) were women, and nine (39.1%) held doctoral degrees. The average years of experience were 15.12 ± 6.73 years, with 5–10 and 11–15 years constituting 60.8% of the total experts. The majority of experts held senior titles, with six (26.0%) having senior titles and twelve (52.2%) having associate senior titles. The basic information on experts is shown in Table 1.

### 3.2. Reliability of Expert Consultation Results

#### 3.2.1. Degree of Activeness of Experts

A total of 24 experts were selected for this study in Sichuan and Chongqing. All 24 questionnaires were returned in the first round, and 23 were returned in the second round, yielding effective response rates of 100.0% and 95.8%, respectively. The structure of the two rounds of expert consultation shows that the experts are highly active.

#### 3.2.2. Authority Coefficient of Experts

In this study, the calculation shows that the Cs for two rounds of expert consultation were 0.94 and 0.92, respectively, while the Ca were 0.96 and 0.95, respectively. Therefore the Cr were 0.950 and 0.936, respectively, both greater than 0.7. This indicates that the Cr of expert opinions across both consultations falls within an acceptable range, affirming their high reliability.

#### 3.2.3. Degree of Coordination and Concentration of Expert Opinions

In the first round of counselling, the CV was 0.041–0.163 and the W was 0.147–0.209; during the second round of counselling, the CV was 0.000–0.157 and the W was 0.144–0.244, as detailed in Table 2. Notably, apart from the importance of the primary indicators, the coordination coefficients of the other indicators improved in the second round compared to the first. Additionally, the significance level of *p* < 0.05 was attained in both rounds, indicating the favorable outcomes of the experts’ consultation and emphasizing the high validity of the developed scale.

### 3.3. Results of the Delphi Survey

**Figure 1:**
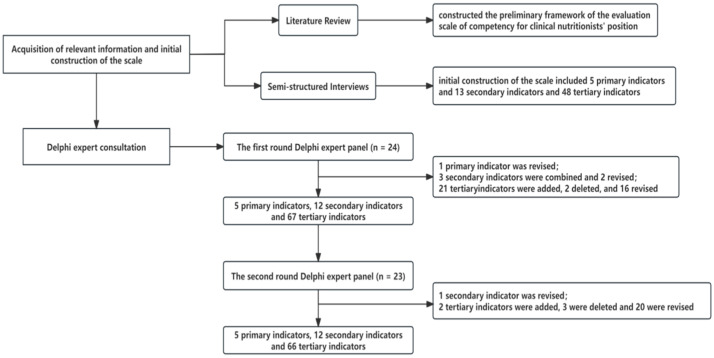
Flowchart for scale construction.

Based on feedback from experts and internal group discussions, several modifications were made to the scale. Following the first round of expert consultation, “scientific research and learning capacities” was revised to “professional development ability” in the primary indicators; “basic nutrition knowledge”, “public nutrition knowledge”, and “clinical nutrition knowledge” were merged into “Nutrition specialty fundamentals of nutrition” in the secondary indicators; and “fundamentals of clinical medicine” and “teaching and training ability” were added. “Nutrition counseling” was revised to “nutrition counseling and education”, and “nutrition screening and assessment” was revised to “nutrition risk screening and status assessment”. Finally, twenty-one tertiary indicators were added, two were deleted, and sixteen were revised, resulting in five primary indicators, twelve secondary indicators, and sixty-seven tertiary indicators. After the second round of expert consultation, “nutritional risk screening and status assessment” was revised to “nutritional screening, assessment and diagnosis” in the secondary indicators, and two tertiary indicators were added, three were deleted, and twenty were revised. The final scale comprised five primary indicators, twelve secondary indicators, and sixty-six tertiary indicators. Figure 1 provides an overview of the entire scale construction process.

Based on the results of the final round of expert consultation and the Saaty scale, calculated using yaahp software, it was concluded that the CR is 0.0013, indicating that the indicators satisfy the consistency criterion [26]. The results of this study show that the indicators at all levels meet the requirements of the consistency test, and the allocation of weights is detailed in Table 3.

### 3.4. Reliability of the Scale

Out of 45 electronic questionnaires collected, 30 were deemed valid. The total number of questionnaire indicators was 66, the calculated Cronbach’s α for scale consistency was 0.984, and Cronbach’s α for the five dimensions was 0.970, 0.978, 0.969, 0.962, and 0.947, respectively, which indicated that the questionnaire had good internal consistency.

### 3.5. Validity of the Scale

In this study, EFA was used to analyze the validity of questionnaire. According to the competency iceberg model, the competency was divided into penetrance characteristics on the iceberg and intrinsic characteristics under the iceberg. The penetrance characteristics included two dimensions of professional theoretical knowledge and professional practical skills, which included five secondary indicators: “1.1. nutrition specialty fundamentals of nutrition”, “1.2. fundamentals of clinical medicine”, “2.1. nutrition counseling and education”, “2.2. nutritional screening, assessment and diagnosis”, and “2.3. nutritional therapy”. The intrinsic characteristics included three dimensions of humanistic practice ability, interpersonal communication ability, and professional development ability, including seven secondary indicators, which were “3.1. professional ethics”, “3.2. medical humanities”, “4.1. communication ability”, “4.2. teamwork ability”, “5.1. self-directed learning ability”, “5.2. scientific research and innovation ability”, and “5.3. teaching and training ability”.

The results showed that the KMO of the penetrance factors and intrinsic factors were 0.782 and 0.812, respectively, and the data passed the Bartlett’s test of sphericity, which indicated that both of them were suitable for factor analysis. A principal component analysis was used to extract the common factors. Five common factors were extracted for the penetrance factors, with a cumulative variance contribution rate of 86.936%, and the results are shown in Table 4. Seven common factors were extracted for the intrinsic factors, with a cumulative variance contribution rate of 94.551%, and the results are shown in Table 5.

## 4. Discussion

Competencies play a crucial role in distinguishing excellence from mediocrity and can be precisely quantified using scientific and rational assessment indicators [28]. The competency evaluation scale can assess the competencies for clinical nutritionists, thereby enhancing motivation and fostering the rapid development of clinical nutrition departments. This research synthesized global insights on nutritionists’ competencies and adapted them to China’s clinical context through expert interviews and a Delphi study, leading to a tailored competency framework and evaluation scale for clinical nutritionists. Ultimately we developed a clinical nutritionist competency evaluation scale with five primary indicators, twelve secondary indicators, and sixty-six tertiary indicators. The Delphi method affirmed the scale’s high reliability and content, with Cronbach’s α indicating strong internal consistency. Factor analysis confirmed its validity, ensuring the scale’s robustness in measuring clinical nutritionists’ competencies.

Utilizing the iceberg model, the competency scale developed in this study assigns weights, in descending order, to the primary indicators: “professional theoretical knowledge” (0.2168), “professional practical skills” (0.2120), “humanistic practice ability” (0.2042), “interpersonal communication ability” (0.2022), and “professional development ability “(0.1649). The first two indicators represent explicit competencies, while the latter three are implicit, underpinning the nuanced and comprehensive nature of clinical nutritionists’ professional skills. The greater weight given to the obvious characteristics indicates that good clinical nutritionists must have solid theoretical professional knowledge and proficient practical skills. Among them, the weight of professional theoretical knowledge is higher than that of professional practical skills, underscoring the importance of possessing a strong theoretical foundation for accurate clinical practice [40]. The professional theoretical knowledge of the program consists of “nutrition specialty fundamentals of nutrition” and “fundamentals of clinical medicine”, with respective weights of 0.1131 and 0.1037. This emphasizes the critical role of nutritional knowledge in clinical settings, highlighting it as a vital competency for clinical nutritionists, which is consistent with Pujia et al. [41]. While the weight difference between nutritional and medical knowledge is minimal, it highlights the necessity for clinical nutritionists to possess a foundational understanding of medicine. This medical knowledge enables them to identify nutrition-related conditions accurately, ensuring appropriate patient care. Inconsistent with the present study, more attention is being paid to improving the nutritional knowledge of medical students and physicians as a way to facilitate disease treatment [42].

Professional practical skills encompass “nutrition counseling and education”, “nutritional screening, assessment, and diagnosis”, and “nutritional therapy”, weighted at 0.0680, 0.0707, and 0.0733, respectively. Nutritional screening, assessment, and diagnosis carry the greatest weight in practice competencies. Proper nutritional risk screening and understanding the patient’s nutritional status are essential for devising a rational nutritional treatment plan to enhance clinical outcomes [43,44]. This is consistent with previous research [45,46]. Nutritional counseling and education are assigned less weight compared to the other two indicators. Throughout the nutritional treatment process, patients receive counseling tailored to their needs from clinical nutritionists, who also disseminate accurate nutritional information, facilitating proper nutritional guidance for patients. When patients receive proper nutritional counseling and education, it improves their nutritional status and reduces complication rates [47]. “Nutritional therapy” is given the most weight because of the specialized nutritional therapy skills that a clinical nutritionist must have in order to ensure that the patient quickly recovers his or her health status. Clinical nutritionists must possess adequate theoretical knowledge and practical skills to excel in their role, aligning with China’s emphasis on the construction of clinical nutrition department requirements [11].

“Humanistic practice ability”, comprising “professional ethics” and “medical humanities”, ranked third among the five primary indicators in the competency evaluation scale, with weights of 0.1088 and 0.0954, respectively. This underscores the vital significance of humanistic practice ability in professional practice. Some studies of clinician competencies have incorporated ethical literacy and humanistic care into physicians’ core competencies [48,49,50]. “Professional ethics” was given a higher weight than “medical humanities” because health workers with strong professionalism can provide quality humanistic care to patients. “Passion for the job” and “adherence to professional standards” are emphasized in professional ethics literacy and represent fundamental competencies for fostering strong professionalism. Moreover, nutritionists, as healthcare professionals, not only use their knowledge and skills to treat patients, but also provide compassionate humanistic care [51,52,53]. This is consistent with the findings of Jin [54] and Wan [27].

“Interpersonal communication ability” comprises “communication ability” and “teamwork ability”, both of which carry equal weight. During patients’ medical treatment, clinical nutritionists require proficient verbal communication skills. Improved communication with patients enhances doctor–patient relationships, reduces disputes, and promotes effective disease management [55,56]. Therefore, “master the verbal communication skills and communicate concisely and clearly” in the tertiary indicators is given more weight. This is consistent with competency studies of clinicians [57] and nurses [58]. Apart from patient and family communication, clinical nutritionists should enhance collaboration with medical professionals within and outside their department to optimize disease treatment. In the study on the competency of general practitioners [59], it is also mentioned that doctors should not only have communication skills but also need to strengthen teamwork in their clinical work.

“Professional development ability” comprises “Self-directed learning ability” (0.0589), “Scientific research and innovation ability” (0.0498), and “Teaching and training ability” (0.0562). This study revealed that “Self-directed learning ability” had the highest weighting in “Professional development ability”. Clinical nutritionists must independently acquire and master cutting-edge clinical nutrition knowledge from both domestic and international sources, and stay informed about the latest developments in nutrition research to keep pace with advancements in the field and enhance clinical nutrition services. However, “Scientific research and innovation ability” has a relatively low weight, and expert interviews revealed that some clinical nutritionists tend to overlook this competency. However, as modern medicine advances, there is an increasing demand for scientific research outcomes in disease treatment. Therefore, clinical nutritionists need to actively engage in scientific research to enhance disease treatment [60,61]. Currently, clinical education in China is predominantly conducted by clinicians [62], and outstanding clinical nutritionists are also expected to possess teaching and training capabilities, applying their clinical expertise to educational settings, which is consistent with the findings of other study of clinicians’ professional development competencies [63].

This study in China marks the first development of a competency evaluation scale for clinical nutritionists, demonstrating its sound validity and reliability. Nonetheless, the scale does possess certain limitations. Firstly, constrained by regional and economic factors, the Delphi consultation was confined to 24 experts, potentially limiting the representativeness and authority of the expert panel. Furthermore, the scale was exclusively developed in the Sichuan and Chongqing regions of China, thereby limiting its representativeness to the southwest region and failing to address the competency requirements of clinical nutritionists nationwide. Finally, the scale lacks real-world clinical application, thus warranting future surveys to gain deeper insights into the competency levels of clinical nutritionists.

## 5. Conclusions

Overall, the competency evaluation scale for clinical nutritionists developed in this study demonstrates high reliability and conforms to the current training requirements for clinical nutrition personnel in China. The tool assesses five dimensions of competencies possessed by clinical nutritionists, providing a comprehensive evaluation. In clinical practice, training programs are developed using this scale to enhance clinical nutritionists’ professional skills. Simultaneously, it serves to evaluate the professional titles of clinical nutritionists, distinguishing between exceptional and average practitioners. The objective recording of clinical nutritionists’ performance during competency assessment is essential. The widespread use of the scale will potentially impact clinical settings. Regular competency assessments aid clinical nutritionists in accurately assessing their abilities, thereby enhancing the quality of clinical nutrition services. Improved clinical service quality correlates with enhanced patient recovery rates. Hence, validating the relevance and adaptability of this scale in real-world settings is crucial for its future application.

## Figures and Tables

**Table 1:** The basic information on experts (*n* = 23).

Characteristics		*n* *	%
Gender	Male	11	47.8
	Female	12	52.2
Age, years	≤40	13	56.5
	41–50	9	39.1
	>50	1	4.4
Education	Undergraduate	4	17.4
	Master	10	43.5
	Doctor	9	39.1
Professional title	Junior	1	4.4
	Intermediate	4	17.4
	Associate senior	12	52.2
	Senior	6	26.0
Field of work	Clinical Medicine	1	4.4
	Clinical Nutrition	20	87.0
	Nutrition and Food Hygiene	2	8.6
Professional experience, years	5–10	7	30.4
	11–15	7	30.4
	16–20	5	21.7
	21–27	4	17.5

* *n*: number.

**Table 2:** The indicators’ coefficients of variation and expert coordination coefficients.

	Indicator	CV				*W*	χ^2^	*p*-Value
		Minimum	Maximum	Mean	SD			
First round	Primary indicators	0.057	0.140	0.102	0.041	0.209	20.019	0.000
	Secondary indicators	0.041	0.163	0.107	0.042	0.193	55.689	0.000
	Tertiary indicators	0.041	0.162	0.112	0.036	0.147	165.730	0.000
Second round	Primary indicators	0.000	0.100	0.063	0.038	0.144	13.259	0.010
	Secondary indicators	0.042	0.149	0.085	0.035	0.244	56.600	0.000
	Tertiary indicators	0.042	0.157	0.083	0.029	0.208	316.244	0.000

**Table 3:** Weight and combination weight of competency evaluation indicators for nutritionists.

Primary Indicators	Weight	Secondary Indicators	Weight	Portfolio Weight	Tertiary Indicators *	Weight	Portfolio Weight
1. Professional theoretical knowledge	0.2168	1.1. Nutrition specialty fundamentals of nutrition	0.5217	0.1131	1.1.1	0.0810	0.0092
					1.1.2	0.0810	0.0092
					1.1.3	0.0810	0.0092
					1.1.4	0.0810	0.0092
					1.1.5	0.0810	0.0092
					1.1.6	0.0776	0.0088
					1.1.7	0.0785	0.0089
					1.1.8	0.0776	0.0088
					1.1.9	0.0666	0.0075
					1.1.10	0.0666	0.0075
					1.1.11	0.0776	0.0088
					1.1.12	0.0728	0.0082
					1.1.13	0.0776	0.0088
		1.2. Fundamentals of clinical medicine	0.4783	0.1037	1.2.1	0.0986	0.0102
					1.2.2	0.1142	0.0118
					1.2.3	0.1111	0.0115
					1.2.4	0.0938	0.0097
					1.2.5	0.1003	0.0104
					1.2.6	0.1003	0.0104
					1.2.7	0.0954	0.0099
					1.2.8	0.0954	0.0099
					1.2.9	0.0954	0.0099
					1.2.10	0.0954	0.0099
2. Professional practical skills	0.2120	2.1. Nutrition counseling and education	0.3209	0.0680	2.1.1	0.2161	0.0147
					2.1.2	0.1989	0.0135
					2.1.3	0.2161	0.0147
					2.1.4	0.2068	0.0140
					2.1.5	0.1622	0.0110
		2.2. Nutritional screening, assessment, and diagnosis	0.3333	0.0707	2.2.1	0.2054	0.0145
					2.2.2	0.2054	0.0145
					2.2.3	0.1987	0.0140
					2.2.4	0.2054	0.0145
					2.2.5	0.1853	0.0131
		2.3. Nutritional therapy	0.3458	0.0733	2.3.1	0.2085	0.0153
					2.3.2	0.2018	0.0148
					2.3.3	0.1884	0.0138
					2.3.4	0.2006	0.0147
					2.3.5	0.2006	0.0147
3. Humanistic practice ability	0.2042	3.1. Professional ethics	0.5326	0.1088	3.1.1	0.2032	0.0221
					3.1.2	0.1953	0.0212
					3.1.3	0.1953	0.0212
					3.1.4	0.2032	0.0221
					3.1.5	0.2032	0.0221
		3.2. Medical humanities	0.4674	0.0954	3.2.1	0.2019	0.0193
					3.2.2	0.2019	0.0193
					3.2.3	0.2019	0.0193
					3.2.4	0.1923	0.0183
					3.2.5	0.2019	0.0193
4. Interpersonal communication ability	0.2022	4.1. Communication ability	0.5000	0.1011	4.1.1	0.3217	0.0325
					4.1.2	0.3565	0.0360
					4.1.3	0.3217	0.0325
		4.2. Teamwork ability	0.5000	0.1011	4.2.1	0.3043	0.0308
					4.2.2	0.2986	0.0302
					4.2.3	0.3971	0.0401
5. Professional development ability	0.1649	5.1. Self-directed learning ability	0.3574	0.0589	5.1.1	0.2550	0.0150
					5.1.2	0.2636	0.0155
					5.1.3	0.2241	0.0132
					5.1.4	0.2573	0.0152
		5.2. Scientific research and innovation ability	0.3018	0.0498	5.2.1	0.2200	0.0109
					5.2.2	0.2076	0.0103
					5.2.3	0.1979	0.0098
					5.2.4	0.1900	0.0095
					5.2.5	0.2200	0.0092
		5.3. Teaching and training ability	0.3408	0.0562	5.3.1	0.3358	0.0189
					5.3.2	0.3234	0.0182
					5.3.3	0.3408	0.0191

*: Detailed tertiary indicators are shown in Supplementary Table S1.

**Table 4:** The composition matrix after the rotation of the penetrance factors.

Tertiary Indicators	Factor 1	Factor 2	Factor 3	Factor 4	Factor 5
1.1.1	0.644				
1.1.2	0.670				
1.1.3	0.753				
1.1.4	0.663		0.646		
1.1.5	0.620		0.619		
1.1.6	0.700				
1.1.7	0.580				
1.1.8	0.645				
1.1.9	0.815				
1.1.10	0.814				
1.1.11	0.813				
1.1.12	0.855				
1.1.13	0.892				
1.2.1		0.868			
1.2.2		0.822			
1.2.3		0.739			
1.2.4		0.915			
1.2.5		0.904			
1.2.6		0.814			
1.2.7		0.712			
1.2.8		0.599		0.576	
1.2.9		0.627			
1.2.10		0.779			
2.1.1			0.809		
2.1.2			0.524 *		0.684
2.1.3	0.596		0.531 *		
2.1.4	0.607		0.574 *		
2.1.5			0.598		
2.2.1				0.838	
2.2.2				0.733	
2.2.3	0.504			0.607	
2.2.4				0.751	
2.2.5				0.710	
2.3.1					0.652
2.3.2					0.637
2.3.3	0.575				0.674
2.3.4					0.876
2.3.5					0.862

*: Failure to meet expected component attribution entries.

**Table 5:** The composition matrix after the rotation of the intrinsic factors.

Tertiary Indicators	Factor 1	Factor 2	Factor 3	Factor 4	Factor 5	Factor 6	Factor 7
3.1.1	0.864						
3.1.2	0.920						
3.1.3	0.925						
3.1.4	0.916						
3.1.5	0.914						
3.2.1		0.843					
3.2.2		0.840					
3.2.3		0.862					
3.2.4		0.855					
3.2.5		0.865					
4.1.1	0.507		0.558				
4.1.2			0.546				
4.1.3	0.569 *						
4.2.1	0.508			0.575			
4.2.2	0.533			0.620			
4.2.3				0.604			
5.1.1					0.717		
5.1.2					0.695		
5.1.3					0.686		
5.1.4					0.809		
5.2.1						0.872	
5.2.2						0.953	
5.2.3						0.915	
5.2.4						0.772	
5.2.5						0.919	
5.3.1							0.888
5.3.2							0.915
5.3.3							0.869

*: Failure to meet expected component attribution entries.

## Data Availability

The raw data supporting the conclusions of this article will be made available by the authors, without undue reservation.

## Supplementary Materials

The following supporting information can be downloaded at: https://www.mdpi.com/article/10.3390/nu16162593/s1, Table S1: The competency evaluation scale for clinical nutritionists in China.
