# Supplementary material for: Development of the Competency Evaluation Scale for Clinical Nutritionists in China: A Delphi Study
Source: Nutrients. 2024 Aug 6;16(16):2593. doi: 10.3390/nu16162593 (PMC11357651; doi:10.3390/nu16162593)
Supplement: Supplementary file 1 [file nutrients-16-02593-s001.zip › nutrients-3118032-supplementary.pdf]

**Table S1.** The competency evaluation scale for clinical nutritionists in China

| Primary indicators | Secondary indicators | Tertiary indicators |
|---|---|---|
| 1.Professional theoretical knowledge | 1.1Nutrition specialty fundamentals of nutrition | 1.1.1 Master the main pathways of substance metabolism in the human body |
| | | 1.1.2 Master the metabolic process of various nutrients in the body and their physiological functions |
| | | 1.1.3 Master the nutrients in various common foods and their nutritional values |
| | | 1.1.4 Master the Chinese dietary reference intakes |
| | | 1.1.5 Master the latest Dietary Guidelines for Chinese Residents |
| | | 1.1.6 Master the dietary guidelines for specific groups (infants, toddlers, preschoolers, adolescents, pregnant women, elderly, etc.) |
| | | 1.1.7 Master the food exchange portion-based recipe preparation and evaluation of recipe calculations |
| | | 1.1.8 Master the clinical manifestations of various nutrient deficiencies and excesses |
| | | 1.1.9 Master the etiology and pathogenesis of nutrition-related diseases and their complications |
| | | 1.1.10 Master the clinical manifestations of nutrition-related diseases and their complications |
| | | 1.1.11 Master the principles of nutritional diagnosis and treatment of nutrition-related diseases and their complications |
| | | 1.1.12 Master the composition and characteristics of nutritional preparations such as formulas for special medical purposes |
| | | 1.1.13 Master the indications and contraindications for enteral and parenteral nutritional support therapy |
| | 1.2Fundamentals of clinical medicine | 1.2.1 Master the fundamentals of anthropotomy |
| | | 1.2.2 Master the fundamentals of physiology |
| | | 1.2.3 Master the fundamentals of biochemistry |
| | | 1.2.4 Master the fundamentals of pathology |
| | | 1.2.5 Master the fundamentals of medical immunology |
| | | 1.2.6 Master the fundamentals of medical microbiology |
| | | 1.2.7 Familiar with the fundamentals of internal medicine |
| | | 1.2.8 Familiar with the fundamentals of surgery |
| | | 1.2.9 Familiar with the fundamentals of obstetrics and gynecology |
| | | 1.2.10 Familiar with the fundamentals of pediatrics |
| 2.Professional | 2.1Nutrition | 2.1.1 Conducting different forms of nutritional counseling |

| | | |
|---|---|---|
| practical skills | counseling and education | (outpatient counseling, follow-up counseling, telephone counseling, internet counseling, etc.)<br>2.1.2 Accurate and efficient collection of clinical history and dietary, exercise and other related health behavior information<br>2.1.3 Personalized nutritional guidance for different individuals<br>2.1.4 Development of different forms of nutrition education (scientific lectures, training, release of scientific videos, etc.)<br>2.1.5 Production of various types of nutrition education materials (courseware, books, audio-visual, etc.) |
| | 2.2Nutritional screening, assessment and diagnosis | 2.2.1 Proficiency in applying a variety of common nutritional risk screening tools (NRS 2002, etc.)<br>2.2.2 Proficiency in applying tools for assessing the nutritional status of different populations (Core Information and Assessment Tools for Nutritional Literacy of Chinese Residents, etc.)<br>2.2.3 Proficiency in applying dietary survey methods to assess the dietary nutritional status of individuals (questioning methods, note-taking methods, weighing methods, chemical analysis)<br>2.2.4 Accurately interprets clinical examination and laboratory biochemical test results for nutrition-related diseases and evaluates the nutritional status of individuals<br>2.2.5 Proficiency in applying anthropometric methods to assess the nutritional status of individuals (body measurements, body composition measurements, etc.) |
| | 2.3Nutritional therapy | 2.3.1 Standardized design of individualized nutritional therapy programs for patients<br>2.3.2 Standardize the design and evaluation of therapeutic dietary recipes for the needs of various disease states<br>2.3.3 Accurate prescribing of nutritional preparations such as formulae for special medical purposes<br>2.3.4 Regulating and guiding the use of nutritional preparations such as food formulas for special medical purposes<br>2.3.5 Dynamic monitoring of patients during the implementation of nutritional therapy and regular post-treatment follow-up and adjustment of the treatment program |
| 3.Humanistic practice ability | 3.1Professional ethics | 3.1.1 Dedicated, motivated and hardworking<br>3.1.2 Treating patients with honesty and integrity<br>3.1.3 Treating patients fairly and equitably<br>3.1.4 Observance of ethics of professional conduct |

| | | |
|---|---|---|
| | | 3.1.5 Compliance with professional rules and regulations |
| | 3.2Medical humanities | 3.2.1 Empathize with patients<br>3.2.2 Protecting patients' privacy<br>3.2.3 Provision of clinical nutrition services based on informed consent<br>3.2.4 Respect the values of patients and their families and rationalize the control of patients' medical costs<br>3.2.5 A sense of humanistic nutrition and respect for food cultures of different genders, religious beliefs and literacy levels |
| 4.Interpersonal communication ability | 4.1Communication ability | 4.1.1 Effective self-regulation of emotions and appropriate expression of emotions to different audiences<br>4.1.2 Master the verbal communication skills and communicate concisely and clearly<br>4.1.3 Effectively clearing doubts, guiding patients and their families, and gaining acceptance |
| | 4.2Teamwork ability | 4.2.1 Sharing of team resources<br>4.2.2 Gain support and recognition from team members<br>4.2.3 Proactively communicating and collaborating with healthcare professionals in all clinical departments for nutritional therapy |
| 5.Professional development ability | 5.1Self-directed learning ability | 5.1.1 Independently review and summarize domestic and international academic literature<br>5.1.2 Proactive learning of cutting-edge clinical nutrition knowledge and guidelines at home and abroad<br>5.1.3 Proactive participation in nutrition academic conferences and practical skills training at home and abroad<br>5.1.4 Self-reflection and summary improvement |
| | 5.2Scientific research and innovation ability | 5.2.1 Innovative thinking in research<br>5.2.2 Standardize the design of research projects related to clinical nutrition<br>5.2.3 Evidence-based implementation of research projects<br>5.2.4 Proficiency in applying statistical software to analyze data<br>5.2.5 Write and publish research project completion reports and academic papers |
| | 5.3Teaching and training ability | 5.3.1 Develop sound instructional objectives and appropriate instructional programs<br>5.3.2 Apply a variety of teaching methods to teach nutrition-related theories and skills<br>5.3.3 Scientific and systematic evaluation of the effectiveness of teaching practice |
